# Chitosan from Crabs (*Scylla serrata*) Represses Hyperlipidemia-Induced Hepato-Renal Dysfunctions in Rats: Modulation of CD43 and p53 Expression

**DOI:** 10.3390/pathophysiology28020015

**Published:** 2021-05-17

**Authors:** Regina Ngozi Ugbaja, Kunle Ogungbemi, Adewale Segun James, Ayodele Peter Folorunsho, Samuel Olanrewaju Abolade, Stella Onajite Ajamikoko, Eniola Olapeju Atayese, Omowunmi Victoria Adedeji

**Affiliations:** 1 Department of Biochemistry, College of Bioscience, Federal University of Agriculture, P.M.B. 2240 Abeokuta, Nigeria; kunleoguns484@gmail.com (K.O.); Whljaymz@gmail.com (A.S.J.); ayodele.peter@pg.funaab.edu.ng (A.P.F.); aboladeolanrewaju@gmail.com (S.O.A.); stellaajams@gmail.com (S.O.A.); atayeseeniola2@gmail.com (E.O.A.); omowunmi.adedeji@yahoo.com (O.V.A.); 2 Department of Chemistry/Biochemistry, Nigerian Stored Product Research Institute, P.M.B. 5044 Ibadan, Nigeria; 3 Biochemistry Program, Department of Chemical Sciences, Faculty of Science, Augustine University, P.M.B. 1010 Ilara-Epe, Nigeria

**Keywords:** chitosan, hyperlipidemia, high-fat diet, p53 and CD43 genes, functional indices

## Abstract

Hepato-renal dysfunctions associated with hyperlipidemia necessitates a continuous search for natural remedies. This study thus evaluated the effect of dietary chitosan on diet-induced hyperlipidemia in rats. A total of 30 male Wistar rats (90 ± 10) g were randomly allotted into six (6) groups (*n* = 5): Normal diet, High-fat diet (HFD), and Normal diet + 5% chitosan. The three other groups received HFD, supplemented with 1%, 3%, and 5% of chitosan. The feeding lasted for 6 weeks, after which the rats were sacrificed. The liver and kidneys were harvested for analyses. Hepatic alanine aminotransferase (ALT), aspartate aminotransferase (AST), alkaline phosphatase (ALP) activity, and renal biomarkers (ALT, AST, urea, and creatinine) were assayed spectrophotometrically. Additionally, expression of hepatic and renal CD43 and p53 was estimated immunohistochemically. The HFD group had elevated bodyweight compared to normal which was reversed in the chitosan-supplemented groups. Hyperlipidemia caused a significant (*p* < 0.05) decrease in the hepatic (AST, ALT, and ALP) and renal (AST and ALT) activities, while renal urea and creatinine increased. Furthermore, the HFD group showed an elevated level of hepatic and renal CD43 while p53 expression decreased. However, groups supplemented with chitosan showed improved hepatic and renal biomarkers, as well as corrected the aberrations in the expressions of p53 and CD43. Conclusively, dietary chitosan inclusion in the diet (between 3% and 5%) could effectively improve kidney and liver functionality via abatement of inflammatory responses.

## 1. Introduction

Hyperlipidemia (HLP) is a family of disorders that are characterized by abnormally high levels of lipids in the blood. It is the contributory factor to the induction and development of atherosclerosis and cardiovascular diseases (CVD) [1,2]. The ever-increasing prevalence of HLP might be responsible for the continuous upsurge in the demographic data of obesity and attendant organ dysfunction worldwide [3]. Indeed, chronic renal failure parallels the occurrence of premature atherosclerosis, and cardiovascular comorbidities and mortalities [4]. Clinical evidence also suggests that atherosclerosis due to HLP predates renal failure [4]. Furthermore, HLP contributes to the onset of fatty liver disease which is prodromal to type-2 diabetes (T2D)-induced hepatocellular damage and mortalities [5]. The hallmarks of liver disease include deranged liver function biomarkers (such as ALT, AST, ALP, and Bilirubin), culminating in non-alcoholic fatty liver disease (NAFLD), and other acute liver failures [5]. Inflammatory responses mediated by the activated blood cells such as macrophages, monocytes, neutrophils, and T-cells represent connects HLP and hepato-renal dysfunctions [3]. Therefore, the mechanism underlying HLP-induced liver and kidney damage might be linked to inflammatory pathways and oxidative stress [6].

CD43, (Sailophorin or Leukosialin) is one of the most common leukocyte transmembrane sialo-glycoproteins that is expressed by inflammatory cells such as monocytes, neutrophils, macrophages, and T-lymphocytes with distinct physiologic functions, such as differentiation, proliferation, and apoptosis [7]. However, its overexpression has also been implicated in different tumors of non-hematopoietic cells and immune-deficiency [8]. Apart from its roles in DNA repair, cell cycle arrest, apoptosis, and oncogene activation [8], p53 has been shown to coordinate intermediary metabolism such as regulation of glycolysis and repression of lipogenesis via the inhibition of sterol regulatory binding protein-1 (SREBP-1). This way, p53 regulates lipid anabolism by compensatory activation of lipid oxidation pathways [9]. Moreover, alterations of p53 expression have been shown to contribute to ageing and associated diseases, cardio-metabolic disorders, atherosclerosis, and cellular hyper-proliferation [9,10].

Chitosan (CTS) is a de-acetylated derivative of chitin, a biopolymer of glucosamine obtained from the exoskeleton of crustaceans such as shrimps, crabs, lobsters, prawns, and cell walls of some fungi [3,11]. The hypolipidemic effect of chitosan has been extensively explored by many scientists [12,13,14]. Further, chitosan has gained much awareness as a pharmacologic material of importance, due to its reported expansive biological activities, including, but not limited to antitumor [15], immune-stimulating [16], anti-allergic [17], cholesterol-lowering, and anti-inflammatory activities [18], and free radical scavenging activities [3]. Despite the extensive studies carried out on the bioactivities of chitosan, there is a dearth of information on the effect of chitosan concerning HLP-induced hepato- and reno-cellular dysfunctions. In addition, the effect of chitosan on hyperlipidemia-invoked dysregulation of p53 and CD43 expression has not been explored. We therefore examine the effects of chitosan on the hepatic and renal biomarkers, as well as the expressions of CD43 and p53 in hyperlipidemic rats in vivo.

## 2. Materials and Methods

### 2.1. Materials

#### 2.1.1. Crab Shell Collection and Preparation of Raw Materials

Crab (*Scylla serrata*) shell wastes (carapace) containing majorly the exoskeleton was collected from Epe market in Lagos, South-West, Nigeria. The crab shells were air-dried and ground to powder using a miller. The powdered crab shell was then transferred to the Chemistry laboratory of Nigerian Stored Product Research Institute (NSPRI), Onireke, Ibadan, Oyo State, Nigeria for the extraction of chitosan and purification.

#### 2.1.2. Extraction of Chitosan

Extraction of chitosan from the powdered carapace samples was done, following the methods of Burrows et al. [19] as succinctly described previously [20]. Briefly, the exoskeleton of the crab was deproteinized using 4% NaOH for 2 h at boiling temperature and demineralized with 1% *v*/*v* HCl overnight. The yield of crude chitin from the exoskeleton was 70%. The chitin was then further deacetylated with 50% NaOH at boiling temperature for 2 h to obtain the chitosan. The yield of chitosan from chitin was 50% with the degree of deacetylation of 85%. The purity (52%) of the chitosan was obtained by precipitation method.

##### Purification of Chitosan

To purify chitosan using the precipitation method, 10 g of the chitosan was dissolved in 1L 2% acetic acid solution contained in a flask and placed on a shaker for 4 h. Then, 0.5 mol.dm^−3^ NaOH was then added and allowed to precipitate. The precipitates were then filtered, washed, and oven-dried at 103 °C for 1 h [21]. The degree of deacetylation (DD) was determined as described by Şen et al. [22]. Chitosan (0.125 g) was dissolved in a 25 cm^3^ aqueous solution of 0.1mol.dm^−3^ hydrochloric acid; this was stirred for about 30 min until the chitosan was dissolved. The solution was titrated with 0.1mol.dm^−3^ NaOH. The degree of deacetylation was calculated as follows:

DD (%) = NH_2_%/9.94%

$$NH_{2}\%=\frac{\left(C_{1}\times V_{1}-C_{2}\times V_{2}\right)\times 0.016}{G\times \left(100-W\right)}\times 100$$

where NH_2_ is the calculated amount of amino group percentage present in the chitosan, 9.94% is the theoretical amount of NH_2_ percentage present in 1 mole of chitosan, C_1_ is HCl concentration in mol.dm^−3^, C_2_ is NaOH concentration in mol.dm^−3^, V_1_ volume of HCl solution in cm^3^, V_2_ is a volume of NaOH solution in cm^3^, and 0.016 is the molecular weight of NH_2_ in 1cm^3^ of 0.1mol.dm^−3^ HCl in g, G is the sample weight in g, W is the water percentage of the sample.

#### 2.1.3. Experimental Animals

A total of 30 male Wistar rats (90 ± 10) g were procured from the Department of Physiology, College of Veterinary Medicine, Federal University of Agriculture, Abeokuta (FUNAAB), South-west Nigeria, and used for the study. The acclimatization period in the experimental location was for two weeks, and rats were fed with commercially available standard rat chow (Top feed) purchased from an animal feed vendor (Agbeloba Farmers’ market, Abeokuta, Ogun State, Nigeria) and clean water *ad libitum* before the commencement of the experiment in the Animal House of the Department of Biochemistry, FUNAAB, Ogun State, Nigeria. They were kept under ambient condition (25 °C, 56% relative humidity, and 12 h day-light cycle). The study was approved by the Departmental Ethical Committee (FUNAABBCH1819-1). The animals were kept in well-ventilated plastic cages at ambient conditions and handled humanely as conformed to the guidelines of the National Research Council [23] on the use of experimental animals.

#### 2.1.4. Experimental Feed Composition

The experimental diet was compounded by reductive inclusion of the chitosan at 1%, 3%, and 5%, respectively as shown in Table 1 below. No macronutrient was removed to adjust the chitosan-supplemented diet. The inclusion was done by adding 1 g, 3 g, and 5 g of the chitosan to 99 g, 97 g, and 95 g of the respective diets to obtain the 1-, 3-, and 5% supplementation respectively.

### 2.2. Methods

#### 2.2.1. Experimental Design

The experimental animals were allotted into six (6) groups using a simple randomization method, each group with five animals. Group 1: Control animals fed with Normal diet, group 2: High-fat diet (HFD) control received HFD only group 3: Normal diet supplemented with 5% chitosan. Groups 4, 5, and 6 were fed with the HFD, and supplemented with 1%, 3%, and 5% chitosan, respectively. The amount of feed provided daily was 100 g. The HFD was offered to the animals in the HFD and HFD + Chitosan groups, whereas the control diet (normal diet was offered to the animals in the normal control and the normal diet + 5% chitosan groups). The animal body weight was taken weekly using a small animal weighing balance.

#### 2.2.2. Sacrifice and Collection of Samples

After six (6) weeks of chitosan-supplemented diets feeding, all the rats were weighed and sacrificed after an overnight fast. The animals were anaesthetized using ketamine [(35 mg/kg)/ xylazine (5 mg/kg)] [24]. The liver and kidney were excised thereafter, washed in cold physiological saline, and frozen until needed for analyses. Some organs were fixed in 4% phosphate-buffered formalin for immunohistochemical analyses.

#### 2.2.3. Sample Preparation and Biochemical Analyses

Alkaline phosphatase (ALP), aspartate aminotransferase (AST), and alanine transaminase (ALT) activities, as well as the urea and creatinine levels were estimated spectrophotometrically (752N 4nm UV-VIS Spectrophotometer, Powai Mumbai, India) according to the manuals from Randox Diagnostic Kits (Crumlin, England, United Kingdom). Other chemicals (NaOH, HCl, Ethanol, NaCl, Phosphate buffer salts) used were of analytical grade, purchased from Sigma Aldrich Ltd. (St. Louis, MO, USA). The biochemical analyses were estimated in the liver and kidney post-nuclear fraction. Post-nuclear fraction of the liver and kidney was prepared by homogenizing 1 g of the tissues in 9 mL of 0.1 M phosphate buffer, pH 7.4, and centrifugation at 3500 rpm for 10 min [3].

#### 2.2.4. Immuno-Histochemical Analyses for CD43 and p53

Paraffin-embedded sections of the liver and kidney tissues were de-paraffinized and rehydrated using graded alcohol concentration [25]. Antigen retrieval was achieved by boiling in 10 mmol/L sodium citrate buffer for 10 min and then steadily cooling to room temperature. Subsequently, the sections were blocked using 3% H_2_O_2_ in methanol for 15 min to inhibit endogenous peroxidase activity. Following washing in phosphate buffer saline (PBS), the sections were incubated overnight at 4 °C with monoclonal rabbit anti-p53 and anti-CD43 primary antibodies (Elabscience, Wuhan, China) at a dilution of 1:40. The sections were incubated with peroxidase-conjugated goat anti-rabbit immunoglobulin G (IgG) at the same dilution of 1:500 for 2 h at 37 °C. The sections were washed in PBS, developed in prepared DAB chromogen solution, lightly counterstained with hematoxylin, dehydrated, mounted, and visualized under the light microscope (Leica DM2700 M, Wetzlar, Germany). The degree of positivity of the proteins (CD43 and p53) in the hepatic and renal cytoplasm was quantified using Image J software [26]. The photomicrograph is a representative slide from five animals, from which 3 slides were quantified.

### 2.3. Statistical Analysis

Data are expressed as mean ± standard error of the mean (SEM). Analyses were done by one–way analysis of variance (ANOVA) using the statistical package for social sciences (SPSS) version 20.0. Where heterogeneity occurred, the means were separated using a Duncan Multiple Range Test (DMRT). Graphs were plotted using GraphPad Prism (Version 5). Immunohistochemistry quantification was done using ImageJ in triplicates.

## 3. Results

### 3.1. Cumulative Weight Gain of the Experimental Animals

There was a significant (*p* < 0.05) increase in the body weight and average weekly weight gain of the animals fed with HFD only when compared with those fed with the normal diet (Table 2). Nevertheless, there was a progressive reduction in the bodyweight of the animals fed with HFD containing a varying level of chitosan (1%, 3%, and 5% respectively). The reduction in the body weight appeared to be lowest in the group fed with HFD + 1% chitosan, which did not differ significantly from the normal + 5% chitosan group.

### 3.2. Effects of Chitosan Supplementation on Renal Biomarkers

**Figure 1:**
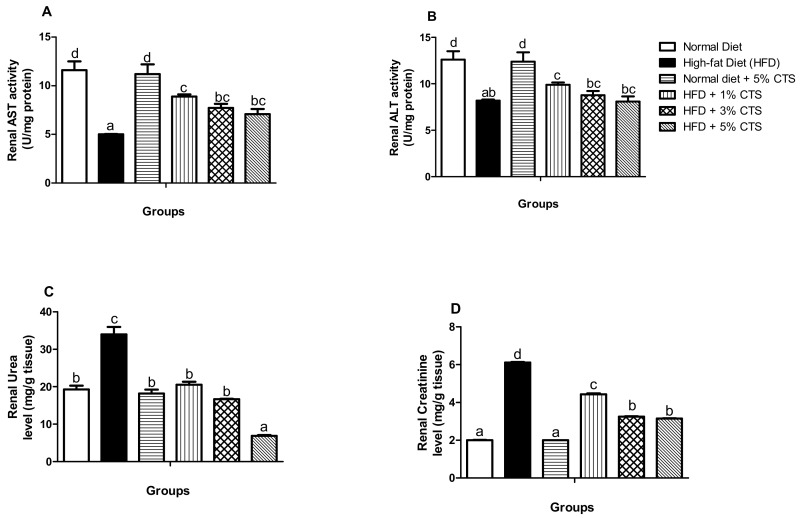
Effects of chitosan supplementation on renal biomarkers of High-fat diet (HFD)-fed rats. Values are expressed as mean ± SEM (*n* = 5). Bars with distinct letters are statistically (*p* < 0.05) different. a–renal AST activity; b–renal ALT activity; c–enal urea level; d–renal creatinine. AST–Aspartate aminotransferase; ALT–Alanine aminotransferase; CTS–chitosan.

The specific activities of AST and ALT, as well as the urea and creatinine levels of rats, maintained on HFD and chitosan are depicted in Figure 1. There were significant (*p* < 0.05) decrements in the activities of AST and ALT in the group fed with HFD alone when compared with the normal diet group. However, the groups supplemented showed gradual improvement in the activities of these enzymes. Interestingly, there appeared to be a hormetic response in the chitosan-treated group, as the 1% chitosan-supplemented group showed more improved activities of AST and ALT. Further, there was no marked difference in the AST and ALT activities between the normal diet and normal diets ± 5% chitosan. Urea level increased significantly (*p* < 0.05) in the untreated HFD group when compared with the normal diet group. Similarly, the renal creatinine level increased in the HFD only group relative to the control. Nevertheless, the group fed with HFD + 5% chitosan showed a lowered urea and creatinine level when compared with the HFD control group. The normal diet + 5% chitosan group did not differ from the normal diet group for both the urea and creatinine levels.

### 3.3. Effects of Chitosan Supplementation on Hepatic Biomarkers

**Figure 2:**
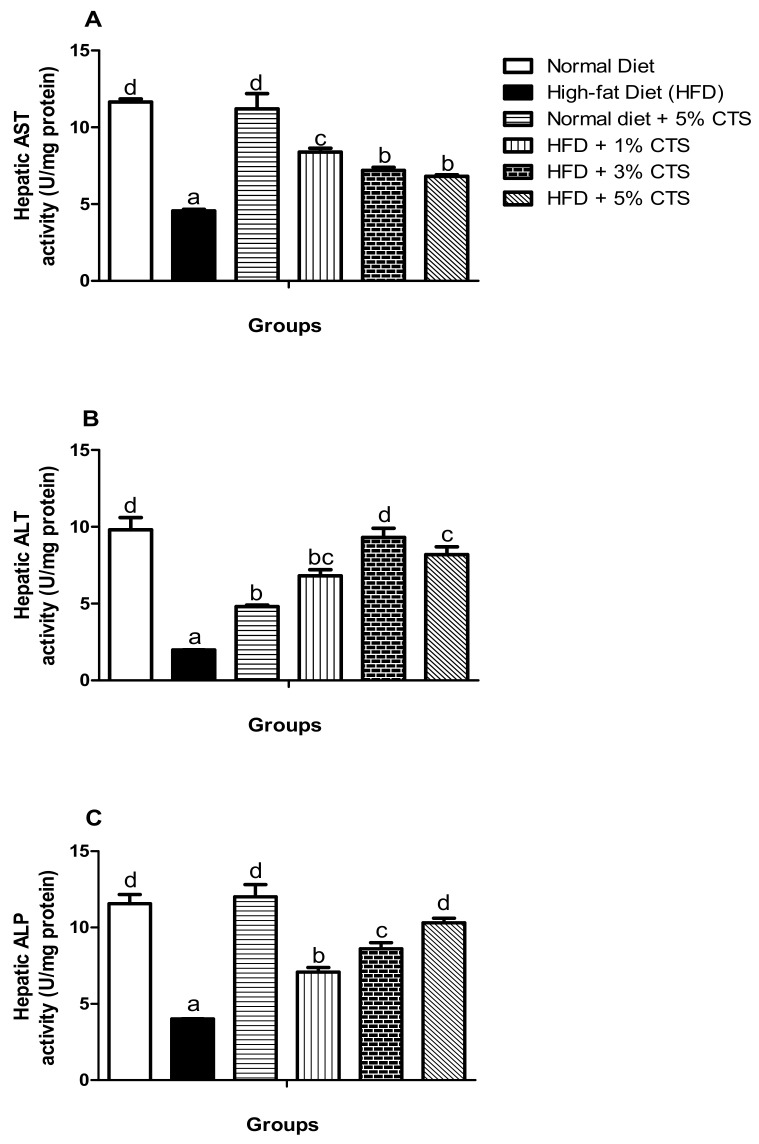
Effects of chitosan supplementation on hepatic biomarkers of High-fat diet (HFD)-fed rats. Values are expressed as mean ± SEM (*n* = 5). Bars with distinct letters are statistically (*p* < 0.05) different. a–Hepatic AST activity; b–Hepatic ALT activity; c–Hepatic ALP. AST–Aspartate aminotransferase; ALT–Alanine aminotransferase; ALP–alkaline phosphatase; CTS–chitosan.

In Figure 2, the hepatic biomarkers (i.e., AST, ALT, and ALP) are shown. Consistently, AST, ALT, and ALP activities were significantly (*p* < 0.05) lowered in the HFD-treated group when matched with the normal diet-fed group. However, the AST activity improved more in the HFD + 1% chitosan group, while no significant difference exists between the 3% and 5% chitosan-supplemented diet groups. Further, there was a dose-dependent increment in the ALP activity of the HFD groups supplemented with chitosan. There was no significant difference in the ALT activity of the rats in the HFD + 3% and 5% groups; otherwise, the increment of the activity is dose-dependent. Strikingly, the hepatic ALT activity decreased significantly in the normal diet + 5% chitosan group compared with the normal diet group.

### 3.4. Effects of Chitosan Supplementation on Hepatic CD43 and p53 Expressions

**Figure 3:**
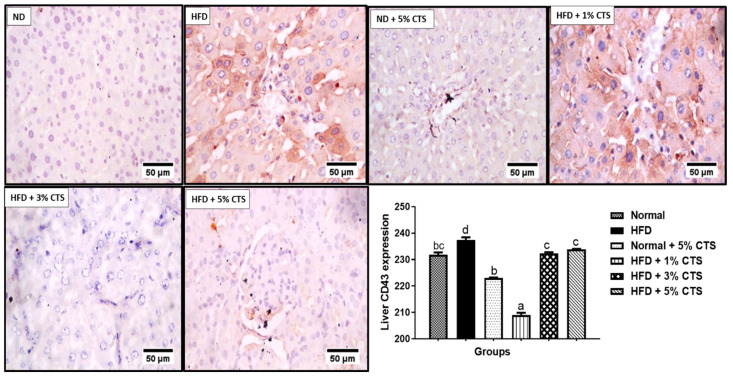
Photomicrograph of immunohistochemical expression of hepatic CD43. Values are expressed as mean ± SEM (*n* = 3). Bars with distinct letters are statistically (*p* < 0.05) different. ND–normal diet; HFD–high-fat diet; CTS–chitosan.

The expression of hepatic CD43 is shown in Figure 3. The expression was markedly (*p* < 0.05) in the HFD group relative to the normal diet group. Nonetheless, the expression was significantly lower in the chitosan-supplemented HFD groups. Interestingly, the group supplemented with 1% chitosan appeared to be the most effective in lowering the CD43 expression. Contrastingly, the expression of p53 decreased significantly in the HFD-fed group when compared with the normal diet group.

**Figure 4:**
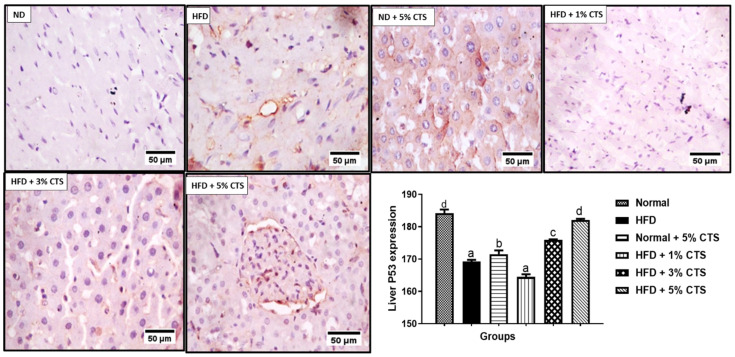
Photomicrograph of immunohistochemical expression of hepatic p53. Values are expressed as mean ± SEM (*n* = 3). Bars with distinct letters are statistically (*p* < 0.05) different. ND–normal diet; HFD–high-fat diet; CTS–chitosan.

However, a dose-dependent increase was observed in the chitosan supplemented group. Indeed, the 5% chitosan normalized, completely the expression of p53 comparable to the normal diet group (Figure 4). The p53 expression was low in the normal + 5% chitosan group when compared with the normal control.

### 3.5. Effects of Chitosan Supplementation on Renal CD43 and p53 Expressions

**Figure 5:**
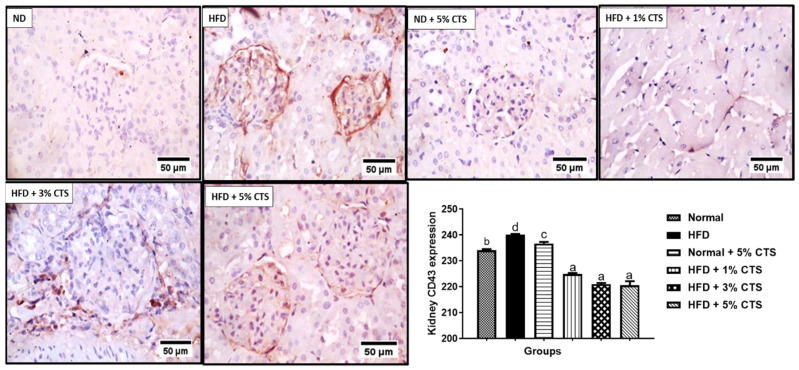
Photomicrograph of immune-histochemical expression of renal CD43. Values are expressed as mean ± SEM (*n* = 3). Bars with distinct letters are statistically (*p* < 0.05) different. ND–normal diet; HFD–high-fat diet; CTS–chitosan.

A significant (*p* < 0.05) increment was observed of renal CD43 expression in rats fed with HFD compared to the normal diet group (Figure 5). However, the HFD + chitosan groups showed considerable decrements in the CD43 positivity. The lowering of CD43 by the different chitosan inclusion appeared to be similar as there was no statistical difference between the groups.

**Figure 6:**
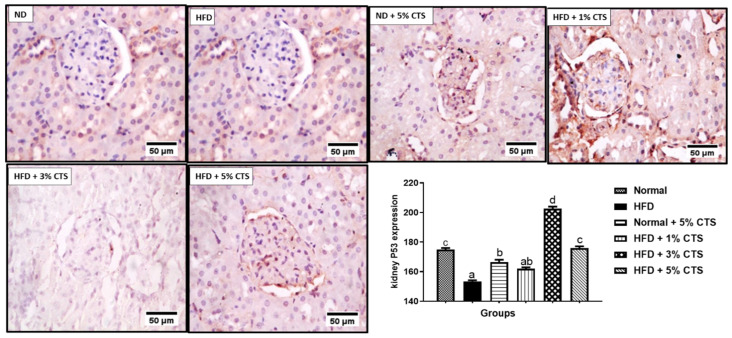
Photomicrograph of immune-histochemical expression of renal p53. Values are expressed as mean ± SEM (*n* = 3). Bars with distinct letters are statistically (*p* < 0.05) different. ND- normal diet; HFD- high-fat diet; CTS- chitosan.

The renal p53 was significantly decreased in the HFD group when compared with the normal diet-fed group (Figure 6). Interestingly, the group fed with HFD + 3% chitosan showed the highest increased level of p53 compared to other groups. Noteworthy, the HFD + 5% chitosan group showed a comparable p53 expression to the normal diet group.

## 4. Discussion

This study provides a possible mechanism underlining the protective in vivo effect of crab chitosan on the liver and kidneys of hyperlipidemic rats. The role of hyperlipidemia in the induction of oxidative stress has been linked to excessive production of ROS [3]. Oxidative stress represents a putative link between the onsets of organ dysfunctions and clinical manifestations [27]. Hyperlipidemia has been shown to worsen liver diseases such as non-alcoholic fatty liver disease (NAFLD), cirrhosis among others, while chronic kidney disease has been linked to hyperlipidemia; both organs dysfunction is inflammation–linked [4,7]. Inflammation is a multi-pathways cellular process involving the transcription factors like NF-κB, and AP-1, cytokines; such as IL-6 and TNF-α, and protein such as the tumor-suppressor gene (p53). Much has been known of p53 as the ‘’guardian of the genome’’ via regulation of cell cycle, cell remodeling, apoptosis among others [28]. Another metabolic activity of p53 has to do with its regulatory function on lipid metabolism and homeostasis. This protein (p53) controls the expression of lipid complexes breakdown and absorption, limits lipogenesis, and inhibits the mevalonate pathway [29]. Recently, the inherent pro-inflammatory role of CD43; a co-stimulatory molecule of the T-cells were identified. It was shown that CD43 possesses the intrinsic ability to activate the NF-κB and cAMP response element-binding protein (CREB) pathways [30]. Furthermore, CD43 has been suggested to coordinate the expression of multiple cytokine genes and act as a chemotactic factor with a pro-adhesive role [30,31]. This study for the first time investigated the possible modulatory effects of chitosan on the expression of hepatic and renal p53 and CD43, as well as hepato-renal functionality of hyperlipidemic rats.

Expectedly, the increased body weight (Table 2) observed in the HFD untreated group is consistent with another study [32], whereby HFD feeding caused an overt increase in body weight of the experimental animal. The increase might be associated with increased food intake [20], as the excessive calorie is accumulated due to HFD feeding culminates to excessive weight, and the attendant hyperlipidemia. However, following supplementation with the chitosan, the increased weight was normalized suggesting the ability of the animals to limit their feed intake due to chitosan inclusion. According to Ogungbemi et al. [20], feeds supplemented with increasing chitosan level had a dose-dependent increment of fiber content as shown in the proximate analyses of the diet. Therefore, a reduction in body weight could also be alluded to controlled feed intake owing to the feeling of satiation and satiety usually associated with a fiber-rich diet [33].

ALT and AST are cytosolic enzymes whose activities have been utilized as an index of hepatic and renal functions. Dysregulation of both enzymes is prodromal to chronic kidney and non-alcoholic liver diseases [33]. These enzymes leak out of the cytoplasm as a direct response to cellular damage into the bloodstream, thereby elevating their plasma activities while the activities reduce in the leaking organ (s) [34]. Our experimental data showed tremendous reductions in the renal activities of AST and ALT with concomitant increments in the urea and creatinine levels in the HFD-untreated group relative to the control animals. This might not be surprising; HFD might causes alteration to renal lipid metabolism by creating an imbalance between lipogenesis and oxidation thereby, inducing a systemic cellular abnormality that might culminate in renal injury and consequent leakage of these enzymes [35,36]. Nevertheless, treatment with chitosan, however, augmented the activities of these enzymes, while reducing the urea and creatinine levels to normal (Figure 1). To the best of our knowledge, we are the first to report the ameliorative effects of chitosan on hyperlipidemia-invoked kidney injury. Akin to AST and ALT, ALP is a marker enzyme for hepatobiliary damage and hepato-necrotic injury which might leak out from the damaged hepatic cells [37]. Accordingly, hepatic AST, ALT, and ALP (Figure 2) decreased in the HFD-untreated group in this study. This might be associated with hyperlipidemia-induced hepatic damage [36]. Regardless, a dose-dependent amelioration was observed in the hepatic ALP activity, while the AST and ALT activities were equally improved in the groups supplemented with chitosan. This observation is consistent with other studies which suggested the hepato-protective role of chitosan [37,38]. Intriguingly, as observed in this study, hepatic ALT reduced significantly in the normal + 5% chitosan group compared to the normal diet group. This observation might indicate two things: (i) 5% inclusion of chitosan might be higher than the therapeutic dose thereby, causing an undesired effect; (ii) when chitosan is not used as a therapeutic approach against disease condition, toxicity might ensue especially at 5% inclusion. The liver, being a major storage organ for chitosan and site for biotransformation of xenobiotics has been said to be susceptible to chitosan toxicity, especially when not metabolized promptly [39]. Accordingly, the in vivo antioxidant and hepato-protective effects of chitosan might be owing to its positively-charged amine group, which limits lipid absorptions in the intestine and also attracts the negatively charged bile components, thereby limiting or inhibiting the accumulation of lipids into the organs [3,40].

In this study, there was downregulation of renal and hepatic p53 expression following HFD feeding relative to the normal diet group. The homeostatic and regulatory role of p53 on intermediary metabolism includes inhibition of lipids biogenesis via a convergent clampdown of de novo fatty acid synthesis and regulation of the mevalonate pathway [28]. The p53 essentially represses the transcription factor; sterol regulatory binding protein-1c (SREBP-1c), which is a master regulator of key lipogenic enzymes, such as fatty acid synthase (FASN), ATP-citrate lyase (ACL), HMG-CoA reductase, and so on [28,41]. Therefore, attenuation of p53 expression in the HFD-fed group might result in over-production of the cellular lipid pools which might also lead to the accumulation of such lipids in the renal and hepatic tissues. As noted earlier, the accumulation of lipids in the renal and hepatic cells are prodromal to hepato-renal dysfunctions [10]. More importantly, the downregulation of p53 might exacerbate inflammation and increases predisposition to cancer and obesity [42]. Experimental evidence suggests that hyper-activation of p53 in the white adipose tissue (WAT) might be due to obesity, and that lowering of p53 in the extra-adipose tissue (such as the liver and kidney) might predispose the organs to accumulation of lipids due to impaired oxidation [43]. The opposite effect observed in our study might indicate a tissue-specific function for p53. Anatomically, the liver and kidney were not meant for lipid storage, therefore, accumulation of lipids in these organs due to energy imbalance might cause organ dysfunction [44]. Nevertheless, our data showed that chitosan possesses the ability to normalize the abatement of p53 expression due to HFD consumption. A dose-dependent increase in p53 expression was observed in the hepatic tissue, while the 3% chitosan-supplemented group showed the highest expression in the kidney. The underplaying playing mechanism behind this observation remains yet enigmatic. However, Lee et al. [45] showed that chitosan nanoparticle activated and upregulated the expression of p53 due to its ability to enter the nucleus to enhance p53 transcription. This might be the underlying mechanism. Interestingly, the group treated with normal diet + 5% showed lower expression of hepatic and renal p53 compared with the normal. These observations were akin to what was observed for hepatic ALT activity, whereby the 5% inclusion in normal diet produced an opposite effect although, in both cases, the undesirable effect was lesser than the HFD group. This might suggest that the inclusion of chitosan up to 5% in a normal diet might produce an undesirable effect, probably due to accumulation and inactivity [39]. Therefore, caution is advised in keeping with this observation.

CD43 expression was elevated in the renal and hepatic tissues of the HFD group that were not treated with chitosan. CD43 is a glycoprotein that is expressed on macrophages and other inflammatory cells with pro-inflammatory cytokines adhesive roles. It has been implicated in every stage of atherosclerotic plaque formation [46,47]. Another implicative role of CD43 upregulation is the inhibition of cholesterol efflux via the ABCA1 and ABCG1 proteins suggesting its pro-hyper-cholesterolemic effect and the attendant atherosclerotic plaque formation. Indeed, hypercholesterolemia initiates and sustains atherosclerosis due to the generation of oxidized low-density lipoproteins (OxLDL) and other metabolic by-products that triggers inflammatory responses [48]. Therefore, upregulation of CD43 expressions in the liver and kidney tissues might be the molecular trigger for the organ’s dysfunction (i.e., liver and kidneys). Nevertheless, our data show that the inclusion of chitosan into the HFD significantly reduced the expression of the hepatic and renal CD43. This observation suggests that crab chitosan might possess an anti-inflammatory effect and the ability to regulate the activity of CD43 and, also, intrinsic ability to inhibit the formation of atherosclerotic plaque in vivo. Indeed, mouse deficient of CD43 (*CD43^−/−^*BMT mice) did not present with atherosclerosis following 16 weeks of high-fat diet consumption relative to the wild type mouse. Suggesting that, upregulation of CD43 is necessary for the formation of atherosclerosis following HFD intake [34,48,49]. Furthermore, CD43 upregulation might exacerbate inflammatory responses and cause attenuation of p53, thereby, increasing predisposition to obesity and cancer [48]. Accordingly, the anti-inflammatory role of chitosan derivatives has been alluded to inhibition of the hyper-proliferation of immune cells, reduction in infiltration of neutrophils and mast cells as well as augmentation of cellular antioxidant systems [3,15]. This study further substantiates the reported pro-inflammatory role of CD43 during hyperlipidemia and suggest chitosan as a possible therapeutic option in the management of the same [15]. Although this study showed a reciprocal modulation of chitosan on the expressions of p53 and CD43, the underlining mechanism remains yet elusive. This might be a focus for further studies as this will shed more light on the implicative roles of p53 and CD43 in hyperlipidemia- induced hepato-renal dysfunction and chitosan as a potential remedy. However, on the account of this study, upregulation of CD43 (a modulator of inflammatory cells trafficking and adhesion) and p53 (regulator of intermediary metabolism and energy homeostasis) suggests that hyperlipidemia might aggravate cellular inflammation and predispose animals to obesity, which correlates positively with hepato-renal damage. Taken together, this study shows that chitosan derived from crabs (at inclusion level between 3% and 5%) possesses hepato-renal protective effects against hyperlipidemia- invoked damages. This is mediated by modulation of kidney and liver biomarkers and down-regulation of pro-inflammatory CD43 expression and upregulation of p53 in rats submitted to high-fat diets. The best modulatory effect of p53 and CD43 was mostly observed between 3% and 5% inclusions when added to HFD. However, inclusion at 5% in the normal diet might produce some undesired effects suggesting that excessive intake of chitosan by healthy people must be with caution.

## Figures and Tables

**Table 1:** Experimental diet composition.

Diet Composition	Ingredients	Normal Diet (g/100 g Diet)	High-Fat Diet (g/100 g Diet)
Carbohydrates	Maize	40	10
	Sucrose	10	10
Lipids	Lard	–	35
	Soya oil	5	5
Protein	Soya	10	10
	Groundnut cake	15	15
	Fish meal	10	10
Fiber	Wheat fiber	5	-
	Mineral mix *	2.5	2.5
	Vitamin mix ^#^	1	1
	Methionine	0.5	0.5
	Lysine	0.5	0.5
	Salt (NaCl)	0.5	0.5

* Mineral mix used was AIN-93-MX. ^#^ Vitamin *mixes* referred to as AIN-93-VX.

**Table 2:** Effect of chitosan (CTS) supplementation on final body weight and average weekly weight gain of rats fed with a high-fat diet (HFD).

Groups	Final Body Weight (g)	Average Weekly Weight Gain (g/week)
Normal control	132.34 ± 1.62 ^b^	7.06 ± 0.28 ^b^
High-fat diet (HFD)	150.06 ± 4.45 ^c^	10.01 ± 0.57 ^c^
Normal + 5% CTS	117.52 ± 1.46 ^a^	4.59 ± 0.18 ^a^
HFD + 1% CTS	116.60 ± 2.07 ^a^	4.43 ± 0.17 ^a^
HFD + 3% CTS	136.42 ± 2.89 ^b^	7.74 ± 0.26 ^b^
HFD + 5% CTS	130.78 ± 1.35 ^b^	6.80 ± 0.21 ^b^

Data are expressed as mean ± SEM (*n* = 5). Values with different superscript (a, b, c) down the column are significantly different (*p* < 0.05).
